# Recurrent Depression and Borderline Personality Disorder in a Patient with Ehlers-Danlos Syndrome

**DOI:** 10.7759/cureus.3760

**Published:** 2018-12-21

**Authors:** Eduardo D Espiridion, Allison Daniel, Joshua R Van Allen

**Affiliations:** 1 Psychiatry, Frederick Memorial Hospital, Frederick, USA; 2 Psychiatry, West Virginia School of Osteopathic Medicine, Lewisburg, USA; 3 Internal Medicine, West Virginia School of Osteopathic Medicine, Lewisburg, USA

**Keywords:** borderline personality disorder, major depressive disorder, ehlers-danlos syndrome (eds)

## Abstract

Ehlers-Danlos syndrome is a group of connective tissue disorders that most commonly causes joint hypermobility, skin hyperextensibility, and tissue fragility. Patients diagnosed with Ehlers-Danlos syndrome (EDS) frequently suffer from chronic pain and other comorbid conditions. In addition, there is an increased incidence of psychiatry conditions in this patient population, such as anxiety, depression, and personality disorders. We present the case of a 25-year-old female who presented to a community hospital with frequent thoughts of suicide and major depression. She was previously diagnosed with Ehlers-Danlos syndrome, borderline personality disorder, and major depressive disorder. The patient was treated with a selective serotonin reuptake inhibitor (SSRI) and gabapentin for pain associated with EDS. However, she was still suicidal and was admitted upon evaluation. Subsequently, she was prescribed lithium in order to augment the effects of the SSRI and reduce her risk of suicide. In addition to pharmacotherapy, patients with these conditions should consider enrolling in some type of therapy.

## Introduction

Ehlers-Danlos syndrome (EDS) is a heterogeneous group of rare, heritable connective tissue disorders occurring in approximately one out of every 5,000 people [1]. These disorders are most commonly characterized by joint hypermobility, skin hyperextensibility, and tissue fragility that can affect multiple organ systems [1]. Chronic widespread pain is a common complaint among these patients that can prove to be both physically and psychologically debilitating. Many patients with an EDS diagnosis have associated psychiatric comorbidities. These conditions include, but are not limited to, major depressive disorder, generalized anxiety disorder, and autism spectrum disorder [2]. A recent study found that patients with EDS are at an increased risk of being diagnosed with depression with a risk ratio (RR) of 3.4 and a 95% confidence interval (CI) of 2.9 - 4.1 [3]. These patients may also be at an increased risk of being diagnosed with a borderline personality disorder (BPD). This disorder is characterized by varying patterns of moods and behaviors [4]. Patients frequently exhibit "splitting", where things are either all good or all bad [3]. Poor impulse control is also a core characteristic of patients with BPD [5]. There is an increase in the number and seriousness of suicide attempts in this patient population [6]. We present a complicated case of a patient with Ehlers-Danlos syndrome with associated major depressive disorder and borderline personality disorder. 

## Case presentation

A 25-year-old female was admitted into a partial psychiatric hospital program after a short stay in the behavioral health unit of a community hospital. She initially presented to the emergency department due to recurrent depression, mood swings, anxiety, and suicidal thoughts/intent. Upon initial evaluation, she reported suicidal thoughts, sad mood, a low energy level, anhedonia, and feelings of worthlessness, helplessness, and hopelessness. She also admitted poor sleep, poor appetite, poor concentration, and recurrent episodes of self-harm. She denied any symptoms of hypomania or mania. However, she did admit to a history of mood swings and anxiety. She had several superficial lacerations on her forearms in multiple stages of healing consistent with self-mutilation. She told staff that she had been “cutting” since she was a teenager and had also tried to intentionally overdose as a teen. The patient had a long history of psychiatric conditions and had multiple prior psychiatric hospitalizations. She denied any history of physical or sexual abuse. She reported a family history of substance abuse, bipolar disorder, and borderline personality disorder. The patient was also diagnosed with Ehlers-Danlos syndrome with symptoms beginning in early childhood.

## Discussion

Studies show that patients diagnosed with Ehlers-Danlos syndrome are at an increased risk of being diagnosed with psychiatric comorbidities [7-8]. A recent study found that patients with joint hypermobility syndrome and EDS had a 4.3 higher risk of being affected by a psychiatric condition [7]. There was also a high prevalence of personality disorders (21%) and Diagnostic and Statistical Manual of Mental Disorders, 5th ed. (DSM-5) Axis-I disorders (38%) in these patients [9]. In addition, these patients are frequently diagnosed with anxiety and depression [2, 8].

A borderline personality disorder is diagnosed by the following criteria from the DSM-5 [9] (a pervasive pattern of instability of interpersonal relationships, self-image and affects, and marked impulsivity beginning by early adulthood and present in a variety of contexts), as indicated by five (or more) of the following:

1. Frantic effort to avoid real or imagined abandonment

2. A pattern of unstable and intense interpersonal relationships characterized by alternating between extremes of ideation and devaluation.

3. Identity disturbance: marked and persistently unstable self-image or sense of self

4. Impulsivity in at least two areas that are potentially self-damaging (e.g., spending, sex, substance abuse, reckless driving, binge eating)

5. Recurrent suicidal behavior, gestures or threats, or self-mutilating behavior

6. Affective instability due to a marked reactivity of mood (e.g., intense episodic dysphoria, irritability, or anxiety usually lasting a few hours and only rarely more than a few days)

7. Chronic feelings of emptiness

8. Inappropriate, intense anger or difficulty controlling anger (e.g., frequent displays of temper, constant anger, recurrent physical fights)

9. Transient, stress-related paranoid ideation or severe disassociate symptoms

The gold standard for treatment of patients with major depressive disorder without psychotic features is pharmacotherapy with depression-focused psychotherapy [10]. The majority of patients are successfully treated with a selective serotonin reuptake inhibitor (SSRI), serotonin-norepinephrine reuptake inhibitor (SNRI), or an atypical anti-depressant [10]. After an initial evaluation, our patient was started on sertraline, 150 mg once daily. However, she did not return to baseline and was still showing characteristic behaviors of BPD. She was also started on gabapentin, 600 mg twice daily, for neuropathic pain associated with EDS. In August 2017, she was started on lithium, 300 mg twice daily, in order to augment the effects of sertraline. BPD is associated with an increased risk of suicide attempts and successes. A recent study has shown that the rate of suicide in patients with BPD is between 8% - 10%, which is greater than the general population [11]. This study also showed that 60% - 70% of patients diagnosed with BPD have already or will make a suicide attempt at some point in their lives [10]. Evidence shows that treating BPD with lithium reduces the patient's risk of suicide and psychopathological features of BPD, such as irritation, anger, and self-harming behavior [12]. The risk of suicide may be reduced by almost 77% [13]. It is not clear whether this decreased risk of suicide is due to a specific “anti-suicidal” property or lithium’s mood stabilization properties [13-14].

Despite treatment, this patient presented to the emergency department due to recurrent depression, anxiety, and suicidal ideations/intent. She had a past history of cutting behavior and an unsuccessful suicide attempt. She was referred to an intensive outpatient therapy program to follow her progress. A promising treatment option for this patient, and many others like her, is dialectical behavior therapy (DBT). DBT is described as an intensive outpatient treatment that focuses on skills training groups, individual psychotherapy, telephone consults, and a therapist consultation team [15]. Patients with BPD enrolled in a DBT have shown a significant decrease in the risk of suicide attempts, psychiatric inpatient hospital treatment, and mood swings [15-16]. DBT for patients with BPD is also useful for decreasing suicide attempts and depression, along with increasing control of anger [17].

## Conclusions

This case demonstrates the importance of managing potential comorbid psychiatric conditions in patients with Ehlers-Danlos syndrome. While managing the chronic widespread pain in these patients is essential, identifying associated psychiatric conditions can easily be overlooked. Recent research has shown that patients with EDS have a higher prevalence of anxiety, depression, and personality disorders. This particular patient’s recurrent depression, borderline personality disorder, and past suicidal behavior made treating these conditions paramount in the management of this patient to preclude potential calamitous complications. Identifying and treating comorbid psychiatric conditions in those with EDS can help these patients with symptoms beyond the quintessential EDS presentation, while subsequently increasing their quality of life.
